# Microsatellite‐based analysis of genetic structure and gene flow of *Mythimna separata* (Walker) (Lepidoptera: Noctuidae) in China

**DOI:** 10.1002/ece3.5799

**Published:** 2019-11-05

**Authors:** Mei‐Mei Li, Bo‐Liao Li, Shi‐Xiong Jiang, Yu‐Wan Zhao, Xiang‐Li Xu, Jun‐Xiang Wu

**Affiliations:** ^1^ State Key Laboratory of Crop Stress Biology in Arid Areas College of Plant Protection Northwest A & F University Yangling China

**Keywords:** gene flow, genetic diversity, microsatellites, *Mythimna separata*, population structure

## Abstract

The oriental armyworm, *Mythimna separata*, is a serious agricultural pest in China. Seasonal and roundtrip migration has recently led to sudden, localized outbreaks and crop losses. To evaluate genetic differentiation between populations in eastern and western China and elucidate gene flow, the genetic structure of 20 natural populations from nine provinces was examined using seven microsatellite markers. The results indicated high genetic diversity. However, little to moderate (0 < *F*_ST_ < 0.15) genetic differentiation was detected, and there was no correlation between genetic distance and geographical distance. Bayesian clustering analysis identified three groups whereas discriminant analysis of principal components identified ten clusters that were considered as two clear‐cut clusters and one admixed group. Gene flow occurred frequently in most population pairs, and an asymmetrical migration rate was detected in several pairwise population comparisons. The bottleneck test showed that few populations had experienced recent bottlenecks. Correspondingly, large‐scale and long‐distance migration of *M. separata* has caused low genetic differentiation and frequent gene exchange. Our findings are important for studying genetic evolution and help to improve predictions of *M. separata* outbreaks in China.

## Graphical abstract

Our study evaluated the population structure, genetic differentiation, and gene flow between geographically distant oriental armyworm populations in eastern and western China using seven microsatellite markers. The results showed that there was little genetic differentiation but high genetic variability and extensive gene flow among populations. We believe that our study makes a significant contribution to the literature because *Mythimna separata* is a serious agricultural pest, and our results will help to improve predictions of its outbreaks in China.

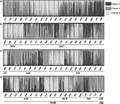

## 1 INTRODUCTION

**Figure 1:**
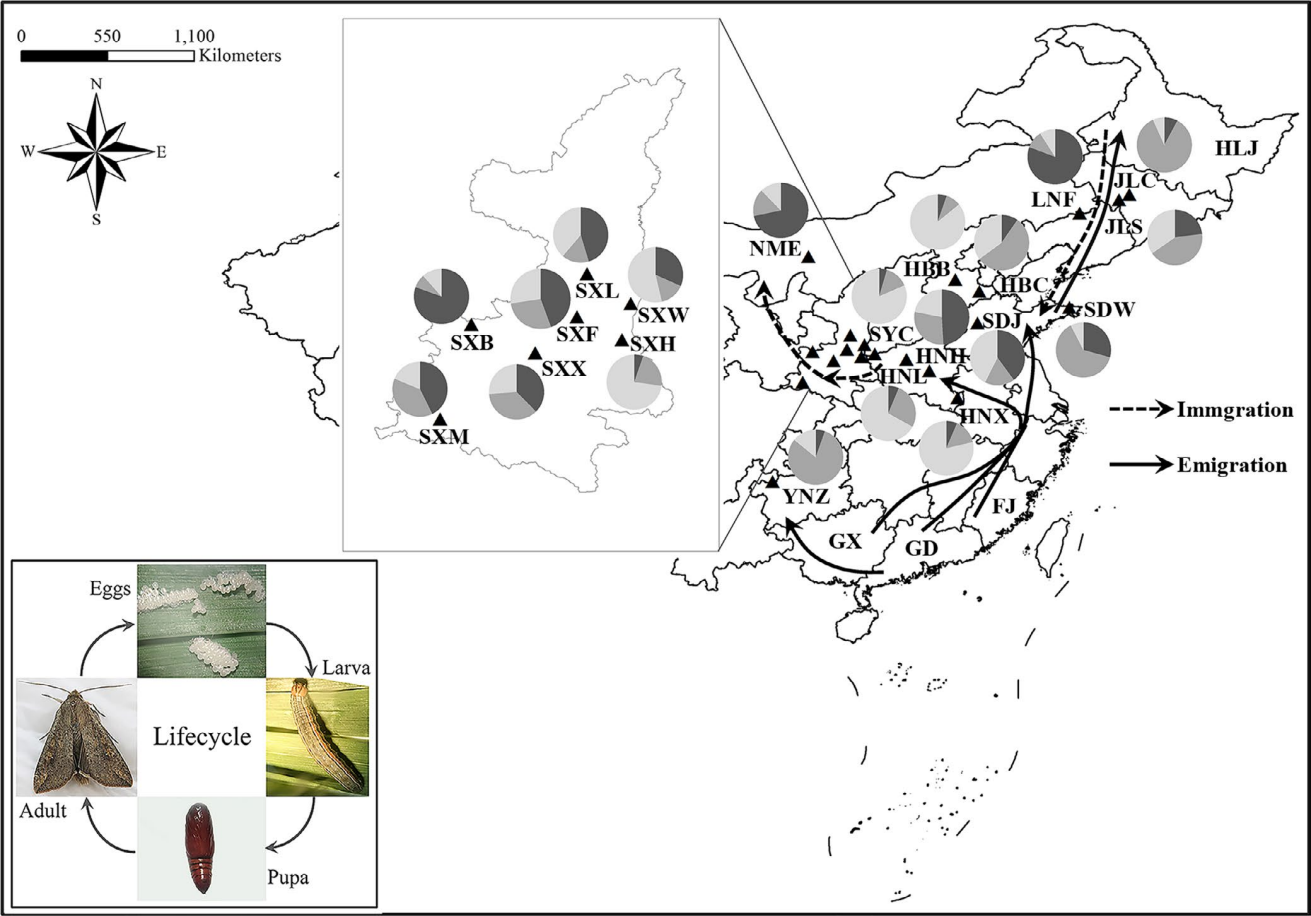
Sampling locations, repartition of microsatellite lineages based on Bayesian clustering analysis using STRUCTURE, and map of the migratory pathways of *Mythimna separata* in China. The inset in bottom left corner shows the life cycle of *M. separata*. Black triangles represent collection sites. Population codes refer to Table 1. HLJ, FJ, GX, and GD represent Heilongjiang, Fujian, Guangxi, and Guangdong provinces, respectively. Dimgray, Darkgray, and Lightgray of pie charts represented Cluster 1, 2, and 3, respectively. The emigration routes (solid arrows) depicted based on the mark‐release‐recapture study of Li et al. (1964), while immigration paths (dotted arrows) delineated in line with gene flow, recent migration rates, and also referring to Feng et al. (2008) and Jiang et al. (2011)

*Mythimna separata* (Walker) (Lepidoptera: Noctuidae) is a common migratory pest in Eastern Asia and some parts of Oceania (Chen, Sun, Wang, Zhai, & Bao, 1995; Farrow & McDonald, 1987). Outbreaks occur intermittently in most regions of China (Zeng, Jiang, & Liu, 2013). It preferentially feeds on Graminaceous crops, such as maize (*Zea mays* L.), wheat (*Triticum aestivum* L.), and rice (*Oryza sativa* L.). The gluttonous behavior of larvae, their short life cycle (Figure 1), and multiple generations per year lead *M. separata* to inflict serious damages and economic losses (Jiang, Zhang, Chen, & Luo, 2014a). Thus, it presents a serious threat to crop production throughout the growing season (Jiang, Jiang, Zhang, Cheng, & Luo, 2014b). The second and third generations of *M. separata* present the most serious infestations, whereas with the third generation becoming the predominant one to produce local infestations in China in recent years (Cheng & Zhao, 2016; Jiang, Zhang, et al., 2014a; Zeng et al., 2013; Zhang et al., 2012).

The armyworm moth undertakes long‐range and multigeneration roundtrip migration between southern and northern China from March to mid‐September each year (Jiang, Luo, Zhang, Sappington, & Hu, 2011; Li, Wang, & Hu, 1964; Zhang, Zhang, Li, Jiang, & Zeng, 2013). This makes it difficulties to predict population dynamics and formulate proper management decisions. Its overwintering boundary indicates that *M. separata* is unable to overwinter in northern China and no diapause period occurs during development (Li, 1993). Therefore, large‐scale outbreaks of *M. separata* in northwestern China are probably attributed to immigration from the eastern regions (Cheng & Zhao, 2016; Zhou et al., 2017). However, seasonal dynamics of armyworm movement between eastern and western China remain unclear.

Based on isozyme analyses, population genetic studies of *M. separata* concluded that oriental armyworm populations formed a large panmictic population from five localities in China (Hao, Li, & Lin, 1992). Researchers further confirmed high genetic variation among individuals of studied populations, and little genetic differentiation was found among geographically distinct populations in China. This is likely due to increased gene flow as a result of their long‐distance migration (Jiang, Luo, & Zhang, 2007; Li, Li, Wu, & Xu, 2018). Furthermore, intersimple sequence repeat (ISSR) analysis implied that highly frequent gene flow among 23 geographically separated armyworm populations from various latitudes prevented genetic differentiation normally caused by genetic drift (Chen et al., 2017). However, their conclusions were unable to reveal population genetic structure and evolutionary history due to limited sampling and the use of molecular markers of low reproducibility or diversity (Chen et al., 2017; Hao et al., 1992; Jiang et al., 2007).

Many studies have been conducted on the migratory flight, northern boundary of overwintering, and evolutionary history of armyworms (Chen et al., 1989, 2017; Hao et al., 1992; Jiang et al., 2007; Li, 1993; Li et al., 1964; Yin, Feng, Cheng, & Cao, 2003; Zhang et al., 2013). However, a comprehensive study of population genetic variation and population structure in China has not been accomplished. The present study analyzed the population structure of 20 geographically distinct populations of the oriental armyworm collected from nine provinces in China, using microsatellite markers. Knowledge about genetic structure among subpopulations, population differentiation, and gene flow of *M. separata* in China is greatly important for determining migration patterns, and thus, the source of outbreaks; it also provides a theoretical framework for prediction and determines control strategies to prevent outbreaks.

## 2 MATERIALS AND METHODS

### 2.1 Sampling collection and DNA extraction

The oriental armyworm larvae samples were collected from maize (*Z. mays*) fields from nine provinces at 20 geographical sites in China (Figure 1; Table 1). A total of 542 armyworms were sampled during the maize‐growing season from May 2016 to August 2017. Each armyworm was collected at a distance of at least 50 m from any other sampled individuals, to avoid sampling siblings. Armyworm larvae were preserved in absolute ethanol and stored at −20°C. Total genomic DNA for genetic analysis was extracted from whole larvae using Biospin insect genomic DNA extraction Kit (Bioer Technology Co., Ltd).

**Table 1:** Sampling information of *Mythimna separata* larvae in China

Collection region (province)	Population code (PC)	Geographical population	Sample size	Generation^a^	Collection time	Latitude/Longitude
Nonoverwintering regions						
Henan (HN)	HNX	Gushi, Xinyang	22	1	3‐May‐16	32.193°N/115.589°E
	HNL	Yichuan, Luoyang	30	3	3‐Aug‐16	34.401°N/112.642°E
	HNH	Linying, Luohe	29	2	22‐Jun‐17	33.739°N/113.966°E
Shanxi (SY)	SYC	Ruicheng, Yuncheng	28	3	8‐Aug‐16	34.694°N/110.840°E
Shaanxi (SX)	SXH	Huayin, Weinan	29	3	5‐Aug‐16	34.546°N/110.075°E
	SXF	Fuping, Weinan	28	2	1‐Jul‐17	34.970°N/109.242°E
	SXM	Mian county, Hanzhong	27	2	2‐Jul‐17	33.075°N/106.707°E
	SXX	Xingping, Xianyang	27	2	3‐Jul‐17	34.303°N/108.468°E
	SXB	Qianyang, Baoji	25	2	4‐Jul‐17	34.832°N/107.284°E
	SXL	Luochuan, Yanan	28	3	4‐Aug‐17	35.771°N/109.429°E
	SXW	Heyang, Weinan	26	3	7‐Aug‐17	35.216°N/110.236°E
Jilin (JL)	JLC	Nanguan, Changchun	26	2	23‐Jun‐17	43.811°N/125.412°E
	JLS	Gongzhuling, Siping	28	2	24‐Jun‐17	43.505°N/124.823°E
Hebei (HB)	HBB	Xinshi, Baoding	22	3	8‐Aug‐17	38.956°N/115.450°E
	HBC	Yunhe, Cangzhou	26	3	10‐Aug‐17	38.283°N/116.820°E
Liaoning (LN)	LNF	Zhangwu, Fuxin	25	3	9‐Aug‐17	42.735°N/122.567°E
Shandong (SD)	SDJ	Changqing, Jinan	29	3	10‐Aug‐17	36.458°N/116.704°E
	SDW	Wangtuan, Weihai	29	3	13‐Aug‐17	37.368°N/121.991°E
Inner mongolia (IM)	NME	Hangjinqi, Ordos	28	3	11‐Aug‐17	40.271°N/107.041°E
Overwintering region						
Yunnan (YN)	YNZ	Zhenxiong, Zhaotong	30	2	12‐Jul‐17	27.408°N/104.980°E

a Presents the local occurrence generation.

### 2.2 PCR amplification and microsatellite genotyping

Seven polymorphic microsatellite loci, A1–91, A3–81, JGTT29, MS1, MS2, MS3, and MS4, developed for *M. separata* were scored for genotyping (Li et al., 2018; Zhang & Zhai, 2008). The three primers used were as follows: a sequence‐specific forward primer linked with M13‐tail at its 5′ end, a sequence‐specific reverse primer, and a FAM or HEX fluorescent‐labeled M13 (−21) primer (Schuelke, 2000). All primers were synthesized by AuGCT Biotechnology. PCR reactions were performed with a total volume of 20 µl, containing 10 µl Golden Star T6 Super PCR Mix (1.1×), 0.4 µl forward primer (10 µM), 1.6 µl fluorescent‐labeled primer (FAM or HEX) and reverse primer (10 µM), respectively, 1.2 µl genomic DNA (20–50 ng/µl), and 5.2 µl ddH_2_O. Amplifications were initiated with a predegeneration 94°C for 2 min, followed by 30 amplification cycles consisting of 94°C for 10 s, 15 s at the primer‐specific annealing temperature, 72°C for 15 s. This was followed by 10 cycles including 94°C for 15 s, 53°C for 20 s, and 72°C for 20 s, and ending with 72°C for 5 min for a final extension. The primer‐specific annealing temperature of each primer was described in previous report (Li et al., 2018). The PCR products of the target band were genotyped by capillary electrophoresis with two‐color fluorescent detection technique at TsingKe Biotech Co., Ltd using an ABI3730XL automatic sequencer (Applied Biosystems). Microsatellite alleles for genotyping were estimated with GeneMarker 2.2 (SoftGenetics LLC) and were confirmed manually.

### 2.3 Genotypic analysis

#### 2.3.1 Levels of genetic diversity

Null allele frequency of each microsatellite locus was estimated using FreeNA (Chapuis & Estoup, 2007) following the Expectation‐maximization (EM) algorithm (Dempster, Laird, & Rubin, 1977), with a significance estimated by 1,000 bootstrap replicates. The ENA (excluding null alleles) correction method (Chapuis & Estoup, 2007) was used to estimate Weir's (1996) global *F*_ST_. The polymorphism information content (*PIC*) was calculated using PowerMarker 3.25 (Lui & Muse, 2005). The mean number of alleles (*Na*), effective alleles number (*Ne*), Shannon's index (*I*), observed heterozygosity (*Ho*), and expected heterozygosity (*He*) were estimated using GenAlEx 6.5 (Peakall & Smouse, 2012) as measures of genetic variability within populations. Gene diversity (*H*), allelic richness (*Ar*), and inbreeding coefficient (*Fis*) within populations were evaluated using Fstat 2.9.3 (Goudet, 1995; Nei, 1973). Deviation from Hardy–Weinberg equilibrium (HWE) for each locus was tested, using Fisher's exact tests implemented in GENEPOP 4.6 (Rousset, 2008). Linkage disequilibrium (LD) between pairs of loci was performed in Arlequin 3.5 with 10,000 permutations (Excoffier & Lischer, 2010).

#### 2.3.2 Genetic structure and population differentiation

The population genetic variance was further analyzed by a global analysis of molecular variance (AMOVA) performed using Arlequin 3.5, and average *F*‐statistics, pairwise differentiation (*F*_ST_), and its significance levels (*p*‐values) were estimated with 1,000 permutations (Excoffier & Lischer, 2010; Weir & Cockerham, 1984). We tested for isolation by distance by regressing pairwise genetic distance (*F*_ST_/(1 − *F*_ST_)) against the natural logarithm of geographical distance (km) across populations for microsatellite loci using the Mantel test (Mantel, 1967) with 1,000 permutations of ZT software package (Bonnet & Van der Peer, 2002). Population structure was analyzed based on Bayesian model‐based clustering using STRUCTURE 2.3.4 with admixture ancestry and correlated allele frequency model (Pritchard, Stephens, & Falush, 2007). Twenty independent runs for each testing genetic clusters (*K*) values which ranged from 1 to 20 were performed with a burn‐in period of 50,000 iterations and 1,000,000 Markov Chain Monte Carlo (MCMC) replicates. The most likely number of clusters (*K*) was determined by considering log‐likelihood values of each *K* and the Delta *K* method described by Evanno, Regnaut, and Goudet (2005), implemented in Structure Harvester (Earl & vonholdt, 2012). CLUMPP 1.1 was used for model averaging of individual ancestry coefficients across the 20 independent runs (Jakobsson & Rosenberg, 2007). Then clusters were visualized using Distruct 1.1 (Rosenberg, 2004). Further, discriminant analysis of principal components (DAPC) was used to identify subpopulations of the species using the Adegenet package for the R 3.5.3 (Jombart, 2008; Jombart, Devillard, & Balloux, 2010; R Core Team, 2019). The number of clusters was assessed using the function *find.clusters*, evaluating a range from 1 to 20. The optimal number of clusters was identified based on the Bayesian information criterion (BIC), as suggested by Jombart et al. (2010). The function *find.clusters* was used to transform original data into principal components (PC), retaining 200 PCs in the analysis. The *dapc* function performed a discriminant analysis using 50 PCs (>80% of variance explained), and nine eigenvalues were retained and examined. The *assign.per.pop* function was used to evaluate the proportions of successful reassignment of individuals to their original clusters. Specifically, we applied the *a.score* and *a.optim.score* functions were used to identify the optimal number of principal components to be retained. Twenty independent runs were executed and the average results were plotted.

#### 2.3.3 Analysis of gene flow and migration rate

Wright's method was employed to estimate gene flow (*N*_e_ *m*) from genetic data based on the relationship *F*_ST_ = 1/(4*N*_e_ *m* + 1) (where *N*_e_ is the effective size of each population and *m* is the migration rate between populations; Slatkin, 1987; Wright, 1931). Bayesian inference for estimating rates of recent migration (over the last several generations) among populations based on multilocus genotypes using BayesAss 3.0 that relies on Markov chain Monte Carlo methods (Wilson & Rannala, 2003).

#### 2.3.4 Bottleneck analysis

The infinite allele model (IAM), the strict stepwise mutation model (SMM) and two‐phase model (TPM) were applied with 10,000 simulation iterations to identify whether populations have experienced bottleneck effects in history under the assumption of mutation–drift equilibrium using BOTTLENECK 1.2.02 (Piry, Luikart, & Cornuet, 2001). A qualitative descriptor of allele frequency distribution (“mode‐shift” indicator), which discriminates between bottlenecked and stable populations, was also utilized (Luikart, Allendorf, Sherwin, Cornuet, & Sherwin, 1998). Under the three models, the standardized differences test was removed from this study because the test is only used when approximately 20 or more polymorphic loci are available. The Wilcoxon signed‐rank test, a more appropriate and powerful method, was applied to detect whether there was a significant heterozygote excess in each population.

## 3 RESULTS

### 3.1 Genetic diversity, Hardy–Weinberg, and linkage testing

A total of 193 alleles were detected over seven microsatellite loci, with a low null allele frequency ranging from 0.029 to 0.185. Therefore, no loci were removed from further genetic analyses. Global *F*_ST_ estimates, with and without ENA correction, were 0.0447 and 0.0453, respectively. The polymorphism information content (*PIC*) ranged from 0.628 to 0.935 (Table 2). For the population level, the mean number of alleles (*Na*) and effective number of alleles (*Ne*) were 9.5 and 5.23, respectively. The estimation for the average of Shannon's index (*I*) was 1.808. The allelic richness (*Ar*) ranged from 6.499 to 11.449 per population, and gene diversity ranged from 0.712 to 0.828. Observed heterozygosity (*Ho*) ranged from 0.580 to 0.702, while expected heterozygosity (*He*) ranged from 0.694 to 0.810. The inbreeding coefficient (*Fis*) within individuals, relative to the rest of their subpopulation, was between 0.097 and 0.297 (Table 3). Additionally, the HBB and YNZ populations possessed the lowest and highest genetic diversity, respectively. Deviations from Hardy–Weinberg equilibrium (HWE) were apparent in six of 20 populations, revealing that these populations tended toward heterozygote excess. Exact tests for departure from HWE across all loci suggested that 95 of the 140 locus‐population combinations deviated significantly (*p* < .05). Linkage disequilibrium presented in 63 of the 147 pairs of microsatellite loci, and no complete linkage of a certain locus was detected.

**Table 2:** Characteristics of each microsatellite loci for 20 *Mythimna separata* populations

Locus (GenBank accession number)	Number of alleles	Frequency of null allele	*F*_IS_	*F*_IT_	*F*_ST_		*PIC*
					Using ENA	Not using ENA	
A1‐91 (FJ896055)	35	0.185	0.393	0.436	0.0463	0.0466	0.872
A3‐81 (FJ896058)	37	0.162	0.335	0.411	0.0912	0.0947	0.889
JGTT29 (FJ896061)	43	0.102	0.206	0.251	0.0359	0.0368	0.935
MS1 (MG594639)	15	0.029	−0.028	0.006	0.0170	0.0164	0.628
MS2 (MG594640)	19	0.045	0.038	0.141	0.0919	0.0914	0.755
MS3 (MG594641)	20	0.049	0.054	0.078	0.0072	0.0067	0.822
MS4 (MG594642)	24	0.115	0.234	0.264	0.0210	0.0175	0.779
Multilocus	193	—	0.176	0.227	0.0447	0.0453	0.811

Abbreviations: *F*_IS_, inbreeding within individuals; *F*_IT_, inbreeding of total population; *F*_ST_, fixation index.

**Table 3:** Population statistics for geographically distinct populations of *Mythimna separata* based on seven microsatellites

PC	*Na*	*Ne*	*I*	*Ar*	*H*	*Ho*	*He*	*Fis*	*p*–HWE
HBB	6.6	3.92	1.440	6.499	0.712	0.643	0.694	0.097	.3198
HBC	10.3	5.20	1.818	9.610	0.785	0.615	0.766	0.217	.0798
HNH	10.1	5.92	1.939	9.504	0.826	0.588	0.808	0.288	.0020**
HNL	8.3	4.12	1.601	7.692	0.739	0.587	0.724	0.205	.0449*
HNX	7.3	4.71	1.678	7.239	0.784	0.678	0.763	0.134	.1282
JLC	10.1	5.54	1.849	9.602	0.794	0.680	0.776	0.142	.1087
JLS	9.4	5.15	1.762	8.746	0.766	0.633	0.750	0.174	.1574
LNF	9.1	4.95	1.768	8.766	0.776	0.625	0.758	0.195	.0876
NME	10.0	5.32	1.866	9.355	0.805	0.664	0.788	0.175	.0469*
SDJ	9.6	4.81	1.779	8.876	0.780	0.629	0.763	0.193	.1166
SDW	10.1	5.39	1.840	9.352	0.793	0.601	0.776	0.242	.0220*
SXB	9.0	5.01	1.777	8.759	0.790	0.702	0.772	0.112	.2396
SXF	9.7	5.07	1.843	9.161	0.803	0.586	0.784	0.271	.1054
SXH	8.0	4.82	1.718	7.589	0.784	0.614	0.768	0.217	.0532
SXL	9.7	5.51	1.851	9.098	0.805	0.628	0.788	0.220	.1697
SXM	10.0	5.78	1.890	9.439	0.813	0.604	0.794	0.257	.1126
SXW	10.7	6.05	1.962	10.181	0.825	0.643	0.805	0.221	.0471*
SXX	10.9	5.82	1.958	10.222	0.820	0.592	0.800	0.278	.0855
SYC	8.9	4.82	1.749	8.368	0.788	0.702	0.772	0.109	.0210*
YNZ	12.6	6.78	2.067	11.449	0.828	0.582	0.810	0.297	.1540
Average	9.5	5.23	1.808	8.975	0.791	0.630	0.773	0.202	.1051

Note

Population codes refer to Table 1.

Abbreviations: *Ar*, allelic richness; *Fis*, inbreeding coefficient; *H*, gene diversity; *He*, expected heterozygosity; *Ho*, observed heterozygosity; *I*, Shannon's index; *Na*, mean number of alleles per locus; *Ne*, effective number of alleles; *p*–HWE, probability value of Hardy–Weinberg equilibrium.

* Significant (*p* < .05) and ** highly significant (*p* < .01) departure from Hardy–Weinberg equilibrium.

### 3.2 Population genetic structure

**Figure 2:**
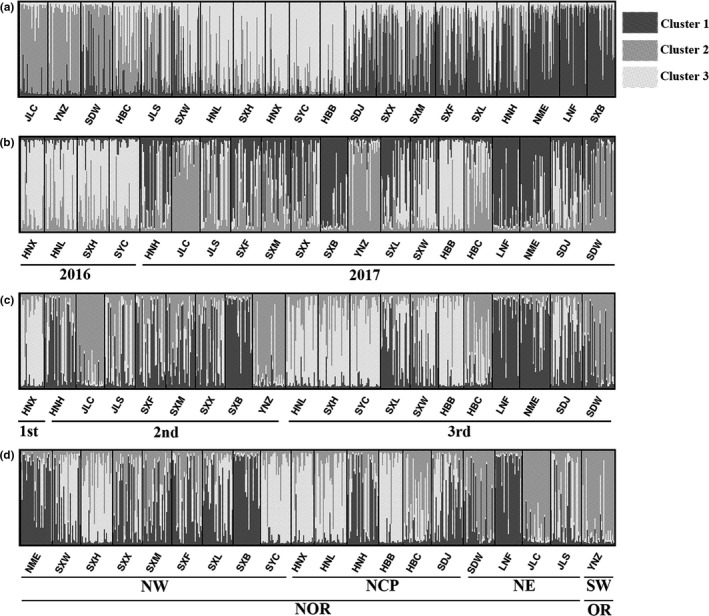
STRUCTURE bar plot assigning 20 populations of *Mythimna separata* to three clusters (*K* = 3). (a) Clustering of the analyzed populations; (b) Collection time: sampled in 2016 and 2017; (c) Generation: 1st, 2nd, and 3rd represented the first, second, and third generation of populations, respectively; (d) Geographical division: NW: northwestern China, NCP: north China plain, NE: northeastern China, SW: southwestern China, NOR and OR represented areas of overwintering and nonoverwintering in China, respectively. The coloring of each bar represents the stacked proportion of assignment to each genetic cluster. Dimgray, Darkgray, and Lightgray of pie charts represented Cluster 1, 2, and 3 respectively

According to the Bayesian analysis performed with STRUCTURE 2.3.4, individuals were grouped into three main clusters (Figure 2a; Figure S1A,B). Although there was a clear signal of population structure, the optimal value of *K* was difficult to determine as no single unifying characteristic was apparent for any of the inferred groups at *K* = 3. Generally, among them, cluster 1 dominated in the LNF and SXB populations, cluster 2 dominated in the JLC and YNZ populations, and cluster 3 dominated in the HBB and SYC populations (cluster with an estimated membership >0.800). However, the other 14 populations showed some level of admixture, and half of them did not completely form their own cluster (<0.500; Tables S1 and S2). Four populations (HNX, HNL, SXH, and SYC) sampled in 2016 shared a similar genetic structure (Figure 2b), which implied that their parent populations came from the same overwintering areas. Populations in the same generation had a similar genetic structure (Figure 2c), indicating the same parental insect sources. The genetic structure of the oriental armyworm populations did not vary significantly per geographic divisions, and the YNZ population from overwintering region had a highly similar genetic structure to that of the JLC population from a nonoverwintering region (Figure 2d).

**Figure 3:**
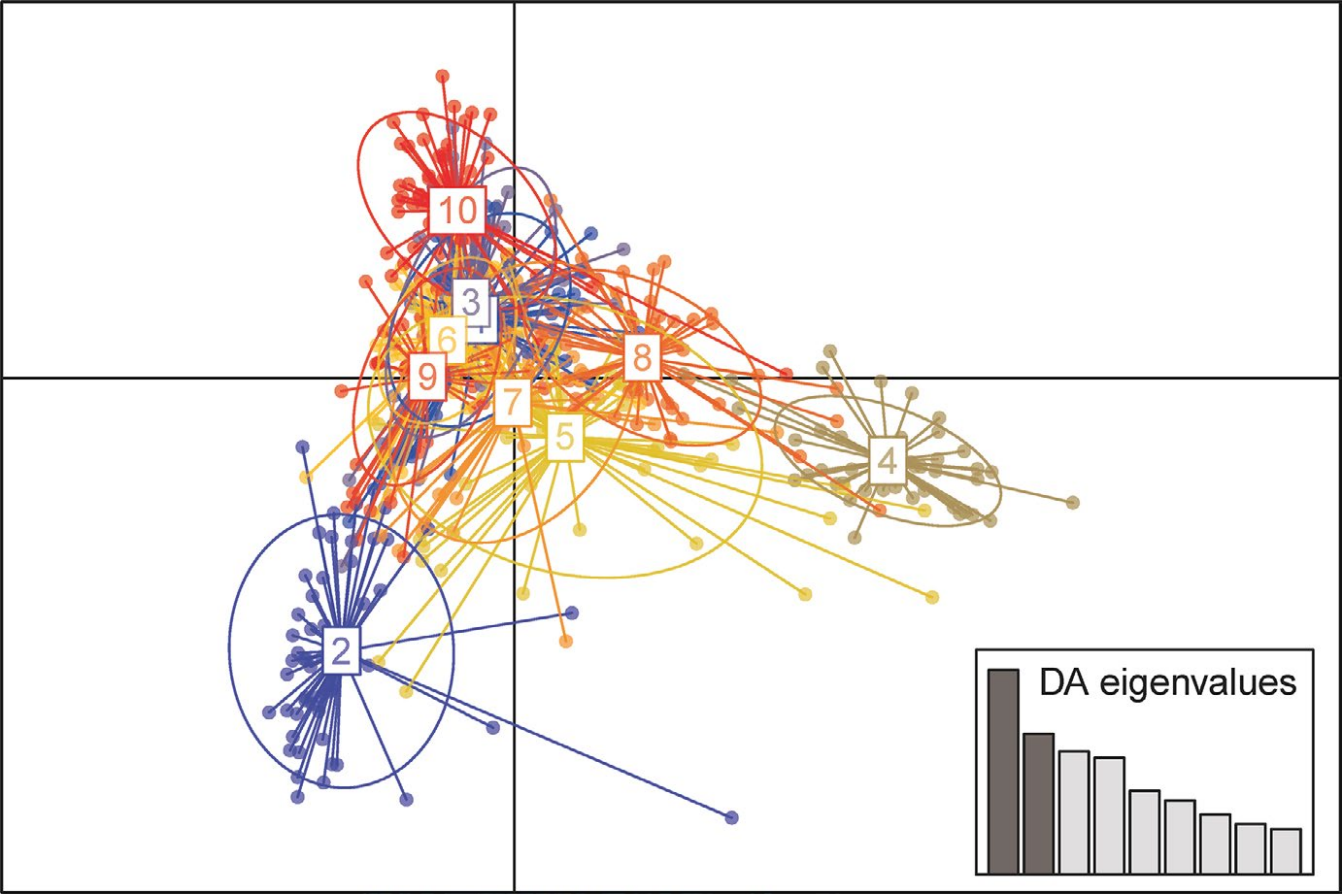
Scatter plots of the discriminant analysis of principal components of the microsatellite data for *Mythimna separata* populations from China. The axes represent the first two linear discriminants (LD). Each circle represents a cluster and each dot represents an individual. Numbers represent the different subpopulations identified by DAPC analysis. DAPC assignment plot based on nine discriminant functions. Eigenvalues of the analysis are displayed in inset

The Bayesian information criteria (BIC) curve in the DAPC analysis supported 10 clusters (Figure S2). Clusters 2, 4 and 10 were clearly differentiated, which indicated that LNF, SDW, and HNL belonged to disparate subgroups. Individuals in the other seven clusters had no distinct division (Figure 3). NME and SXB had similar genetic structure, and SDJ and HBB also had similar genetic structure but were differed from the structure of NME and SXB (Table S3). Inspection of the DAPC plot also revealed the presence of a genetic structure within these populations and was more likely to divide them into three groups (Figure 3). Per Bayesian clustering, no clear associations between the groups were identified using DAPC.

Combining results from Bayesian analysis in STRUCTURE and DAPC, individuals from NME and SXB to be genetically differentiated from LNF, as well as HBC (Figures 2 and 3). Although the four populations from 2016 were in various subgroups, they were weakly differentiated. Individuals from the JLC and YNZ populations belonged to different clusters and were partial overlapped in DAPC analysis despite they divided into multiple genetic clusters, while they had a similar genetic partitioning in Bayesian analysis. Genetic clustering of individuals in each population presented mixed ancestry (Figure 2 and 3), which led to multiple genetic clusters found in armyworm populations. Consequently, these armyworm populations were considered to originate from three differentiated subpopulations.

### 3.3 Population genetic differentiation

The global analysis of molecular variance (AMOVA) test results attributed 4.54%, 19.72%, and 75.74% of the variation to differences among populations, among individuals within populations, and within individuals, respectively, based on the pairwise differences distance method (Table 4). The global *F*_ST_ value, calculated across all populations and loci, was 0.045 (*p* < .001). It is generally assumed that *F*_ST_ < 0.05, 0.05 < *F*_ST_ <0.15, 0.15 < *F*_ST_ <0.25, and *F*_ST_ > 0.25 exhibit little, moderate, significant, and high levels of genetic differentiation, respectively (Wright, 1978). The pairwise *F*_ST_ values (Table S4) were low to moderate, with a maximum between SXB and HBB (0.1129), which indicated a high level of movement among populations. Overall, 58.42% of populations demonstrated little genetic differentiation (*F*_ST_ < 0.05). With the exception of SDJ population, moderate differentiation presented between the HBB population and 18 the other populations (*F*_ST_ > 0.05). The findings illustrated that genetic divergences in the armyworm were mainly between individuals and that gene flow among populations in China was not limited.

**Table 4:** Analysis of molecular variance on seven microsatellite loci in geographically distinct populations of *Mythimna separata*

Global analysis					
Source of variation	*df*	Sum of squares	Variance components	Percentage variation	*F*‐statistics
Among populations	19	196.966	0.13180 Va	4.54	*F*_IS_ = 0.207 *p* < .001
Among individuals within populations	522	1,717.879	0.57237 Vb	19.72	*F*_ST_ = 0.045 *p* < .001
Within individuals	542	1,176.500	2.19894 Vc	75.74	*F*_IT_ = 0.243 *p* < .001
Total	1,083	3,091.345	2.90311		

**Figure 4:**
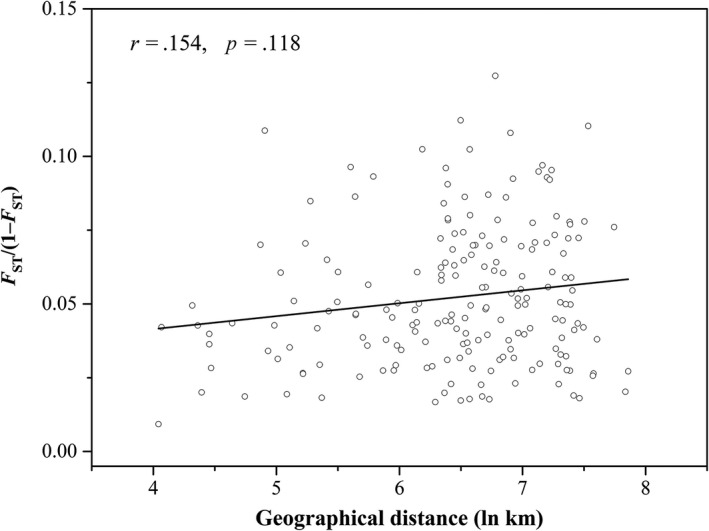
Scatter plots of pairwise genetic distance (*F*_ST_/(1 − *F*_ST_)) against the natural logarithm of geographical distance (km) for pairwise population comparisons

The Mantel test revealed a positive but not significant correlation (*r* = .154, *p* = .118) between the *F*_ST_/(1 − *F*_ST_) ratio for pairs of populations against the natural logarithm‐transformed geographical distance (Tables S4 and S5), demonstrating that the population genetic structure of *M. separata* in China did not conform to the isolation by distance model (Figure 4). That is, no correlation between pairwise genetic distance and geographical distance of armyworm populations was evidenced. Furthermore, geographical distance did not reflect a barrier to gene flow.

### 3.4 Gene flow and migration rate

Observations of the effective number of migrants per generation (*N*_e_ *m*) of several population pairs were greater than 4 (Table S5), indicating that active dispersal ability among those populations was not limited. It is generally assumed that *N*_e_ *m* equal to 1 is sufficient to bring the two populations genetically closer to each other, and if *N*_e_ *m* > 4, then local populations belong to one panmictic population (Wright, 1938). The value of *N*_e_ *m* between SXH and SYC populations was 26.9239, indicating extremely frequent gene exchange or perhaps the same parent population. The gene flow analysis revealed a long‐distance dispersal event (especially in SXX/YNZ and JLS/SDJ) during a period of large‐scale migration from June to August 2017. Theoretically, *N*_e_ *m* also assumes that populations are in migration‐drift equilibrium, which occurs when the increase in genetic differentiation caused by drift is equal to the decrease in genetic differentiation caused by gene flow (Joanna, Heather, & Stephen, 2011). Estimates of gene flow based on microsatellite data analysis indicated high levels of gene flow and the absence of genetic differentiation among population pairs.

The results showed that the recent migration rates ranged from 0.0059 to 0.1817, and asymmetric gene flow existed in some interpopulations, especially JLS, SXH, and SXX (Table S6). The rate of self‐distribution within the SXH population was the highest (0.8613), while that of the SDJ population was the lowest (0.6733). In 2016, the analysis for migration direction across four populations (HNL, HNX, SXH, and SYC) confirmed that the Henan populations (HN) migrated westward to Shaanxi and Shanxi regions (SX and SY) of China. Moreover, Yunnan populations (YN) migrated eastward or northward to Shandong regions (SD) and near Jilin (JL); the Henan population (HN) migrated westward to Shaanxi (SX) and farther northward to Inner Mongolia (IM) in 2017 (Table 1; Figure 1).

### 3.5 Population demography

An attempt to test heterozygote excess was inconsistent under the IAM, TPM, and SMM models (Table 5). The bottleneck test indicated that heterozygote excess was not significant (*p* > .05) in any populations under the SMM model. Conversely, heterozygote excess of most populations was apparent (*p* < .05) under the IAM model, except HBC and JLS populations, elucidating that those populations experienced a reduction in effective size. The discrepancy between IAM and SMM might result from either a small sample size or an influence of compound and complex loci. Under TPM model, significant heterozygote excess (*p* < .05) was detected in three of the populations (HBB, HNX, and SXH), indicating that these populations deviated from mutation–drift equilibrium and might have recently undergone a genetic bottleneck; allele frequency presented a normal L‐shaped distribution (Table 5).

**Table 5:** Wilcoxon's signed‐rank test for heterozygote excess with probability values based on three models (infinite allele model [IAM], two‐phase model [TPM], and strict stepwise mutation model [SMM]) and distribution of allelic frequencies

PC‐sample size	Heterozygosity excess *p*‐values			Distribution of allelic frequencies
	IAM	TPM	SMM	
HBB‐22	**.00391**	**.03906**	.96094	Normal L‐shaped
HBC‐26	.05469	.98828	1.00000	
HNH‐29	**.00391**	.05469	.98047	
HNL‐30	**.00781**	.71094	.98047	
HNX‐22	**.00391**	**.03906**	.59375	
JLC‐26	**.02734**	.65625	1.00000	
JLS‐28	.05469	.65625	.97266	
LNF‐25	**.01172**	.76563	.99609	
NME‐28	**.01172**	.53125	1.00000	
SDJ‐29	**.00781**	.81250	1.00000	
SDW‐29	**.02734**	.53125	.98047	
SXB‐25	**.01953**	.53125	.98828	
SXF‐28	**.03906**	.71094	.98047	
SXH‐29	**.00391**	**.02734**	.71094	
SXL‐28	**.00391**	.28906	.97266	
SXM‐27	**.00391**	.18750	.81250	
SXW‐26	**.00781**	.46875	.99219	
SXX‐27	**.01953**	.76563	1.00000	
SYC‐28	**.00781**	.34375	.98828	
YNZ‐30	**.00391**	.81250	1.00000	

Note

Population codes are given in Table 1; bold indicates significant at *p* < .05.

## 4 DISCUSSION

### 4.1 Genetic diversity

In this study, 193 alleles were found and *M. separata* had a higher genetic diversity based on seven polymorphic microsatellites with low null allele frequency (Tables 2 and 3). The *Fis* values for all populations were greater than zero, indicating a heterozygote deficiency, which caused a deviation from HWE (Table 3). Overall, *Ho* values for all populations were lower than *He*, which might be attributed to null alleles, Wahlund effect, and the population not being in HWE (Joanna et al., 2011). There was no complete linkage of any locus, indicating that the selected loci were evenly distributed in the genome of armyworm and were relatively independent in the process of generational transmission (Torriani, Mazzi, Hein, & Dorn, 2010). The genetic indices showed that the level of genetic diversity in most populations was similar. Higher genetic diversity was observed in the second and third generations, except the HBB population. Furthermore, the YNZ population from the overwintering region exhibited a higher genetic diversity than others from nonoverwintering areas. Abundant genetic diversity was shown to be the genetic basis for its strong adaptive potential (Chen et al., 2017; Wang, Yang, Lu, Zhou, & Wu, 2017), which aids in understanding why *M. separata* localized, large‐scale outbreaks occurred in China in recent decades (Cheng & Zhao, 2016; Jiang et al., 2011; Jiang, Zhang, et al., 2014a; Zeng et al., 2013).

### 4.2 Genetic structure

Bayesian clustering and DAPC analyses (Figures 2 and 3) suggested that the populations were mostly clustered into three groups. All populations were admixtures of these clusters, which could be only explained by the highly migratory ability of the armyworm gave rise to extensive gene flow. Although genetic structure appeared irrelevant to both geographical divisions and occurrence generations of *M. separata* populations throughout China, four populations from 2016 had a similar genetic partitioning which grouped in cluster 3 (Figure 2). As in Coulon et al. (2008), individuals with maximum inferred ancestry <0.6 were not assigned to any group and were considered to exhibit admixed assignation. That was, nine of the populations could not be assigned to any cluster (Table S1). Furthermore, DAPC confirmed that there was little genetic distinction among clusters 1, 3, and 5–9, which mainly contained 15 populations excluding: HNL, LNF, SDW, SXF, and SXW (Figure 3). Based on these results, we inferred that individuals from 20 geographic populations in the same cluster had the same immigration source; they likely shared a common gene pool in the past. AMOVA analysis found low to moderate genetic differentiation (global *F*_ST_ = 0.045, *p* < .001) based on *F*_ST_ among populations. Our results indicated that migratory dispersal led to differences in population structure but they still belonged to a panmictic population owing to their high capacity for long‐distance migration.

### 4.3 Genetic differentiation and migration

There was no significant correlation between geographical distance and genetic distance among any of the 20 populations (Figure 4). Our estimates (Table S5) suggested extensive gene flow (*N*_e_ *m* > 1.0), between pairwise populations, even between most adjacent populations of *M. separata*. This might be owing to its migratory behavior and nongeographical isolation. Therefore, a high gene flow of armyworms (Table S5) had a homogenizing effect on the genetic variation over geographic populations, counteracting random drift, selection, and mutation (Sun, Li, Yang, & Hong, 2012). Notably, the analyses of 20 geographically separated *M. separata* populations showed little to moderate genetic differentiation and a high gene flow (Tables S4 and S5). Insect species might be divided into genetically diverse populations that cover large and heterogeneous geographical regions with limited gene flow (Chen, Tan, Liu, Wang, & Li, 2000). In contrast, geographically distinct armyworm populations did not exhibit genetic differentiation, and isolation by distance was absent due to extensive gene flow, which further validated our findings. Previous research elucidated that frequent genetic exchange occurred between widely separated populations of *Helicoverpa zea* and that these populations did not show any signs of differentiation based on location (Perera & Blanco, 2011). The lack of genetic differentiation more likely reflected genetic mixing between eastern and western North American monarchs, *Danaus plexippus* (Lyons et al., 2012). Therefore, migratory insects form a large panmictic population experience enhanced gene flow because of genetic exchange, migratory flight, and long‐range dispersal. Additionally, the present study articulated that there was no population differentiation caused by genetic drift and no clear evidence for isolation by distance, which is consistent with other studies of this insect (Chen et al., 2017; Li et al., 2018).

The migration rates in BayesAss suggested that migration was directional (Table S6). Thus, the parent populations of the northwestern outbreak areas were from northeastern and/or southern overwintering regions. Specifically, in northwestern China, insect source of the second generation mostly came from Henan, while the third generation migrated from northern China and/or Hebei. The results of migration rates were mainly consistent with pairwise *F*_ST_ and *N*_e_ *m* values, that was, little genetic differentiation corresponded to extensive gene flow and large migration rates (Tables S4–S6). Furthermore, the Wilcoxon signed‐rank test of heterozygote excess (Table 5) elucidated that the studied populations were not subjected to recent bottlenecks; hence, genetic diversity across populations was not affected.

Generally, there are two large‐scale and long‐distance migrations, that is, seasonal and roundtrip flight between southern and northern China (Li et al., 1964). According to the results of genetic structure, gene exchange, and migration rate, the oriental armyworm exhibited a particular flight pathway between eastern and western China. Armyworm moths in western China were from overwintering regions that migrated northward to HN in mid‐April. Its offspring adults that migrated northwestward to the central SX plain, SY, and even possibly continued migrating northward to IM or migrated to north China plain (HB) and northeastern region (JL, LN, and HLJ) from late May to early June. In late July, most adults in northeastern region migrated southward to HB/SD or so far as to Henan. From late August, the armyworm adults in the northwestern areas (SX, SY, and IM) migrated southward back to overwintering regions (FJ, GD, and GX; Table 1; Figure 1). This migratory pathway agrees with the seasonal north–south migration pattern, based on mark‐release‐recapture data and trajectory simulation analysis of northeast populations from Shanxi, southern HB, northern HN, and most of the SD regions (Jiang et al., 2011; Li et al., 1964). The next generation migrated through the central and western regions of HB and IM, and some flew directly from the SD Peninsula across the Bohai Sea to the north of LN and center of JL by radar observation (Chen et al., 1989; Zhang et al., 2013). It was further observed that autumn migration of *M. separata* that originated in northeastern China (i.e., LN, JL, HLJ, and part of IM regions) could immigrate into east–central China (SD) and subsequently to southern China (i.e., FJ, GD, and GX; Table 1; Figure 1) within a week for overwintering (Feng et al., 2008). Considering the lack of data on the overwintering generation, more research is needed to elucidate the migratory patterns in the western areas, in order to better predict and prevent outbreaks.

## 5 CONCLUSIONS

Microsatellite markers elucidated higher genetic diversity of the second and the third generations of *M. separata*. Our analysis showed that little population structure, the lack of genetic differentiation and its extensive gene flow among populations in China, which in consistent with the fact that populations of this species undertakes a long‐distance and large‐scale migration. In addition, bottleneck effects and genetic drift have not yet affected the genetic differentiation of *M. separata*. Finally, sampling including all generations occurring in a year across its entire distribution in China is required to further confirm genetic structure of populations, which reflects the interaction of genetic drift, mutation, and migration, with influences from life history. Greater knowledge on the genetic variation in natural populations of *M. separata* will influence regional outbreak patterns in China by ensuring proper pest prediction and control in advance.

## CONFLICT OF INTEREST

None declared.

## AUTHORS CONTRIBUTION

X.L.X. and J.X.W. conceived the study; M.M.L., S.X.J. and Y.W.Z. participated in collecting the biological samples; and M.M.L. and B.L.L. analyzed the data and wrote the manuscript. All authors contributed to editing the manuscript.

## Data Availability

Microsatellite data file has been accessioned at Dryad (http://datadryad.org) under the title: “Microsatellite‐based analysis of genetic structure and gene flow of *Mythimna separata* (Walker) (Lepidoptera: Noctuidae) in China” (https://doi.org/10.5061/dryad.5tb2rbp0n).
