# Supplementary figures and images for: Microsatellite‐based analysis of genetic structure and gene flow of Mythimna separata (Walker) (Lepidoptera: Noctuidae) in China
Source: Ecol Evol. 2019 Nov 5;9(23):13426–37. doi: 10.1002/ece3.5799 (PMC6912921; doi:10.1002/ece3.5799)

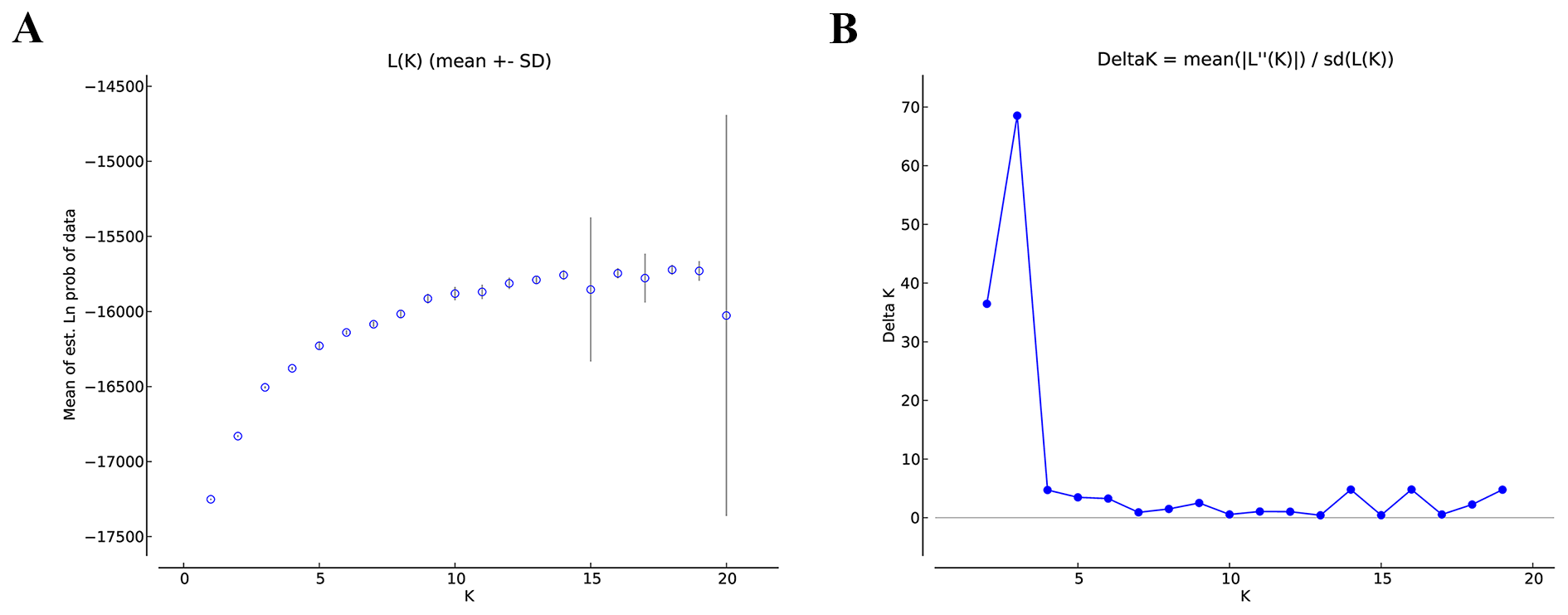

Supplement: Supplementary file 1 [file ECE3-9-13426-s001.tiff]

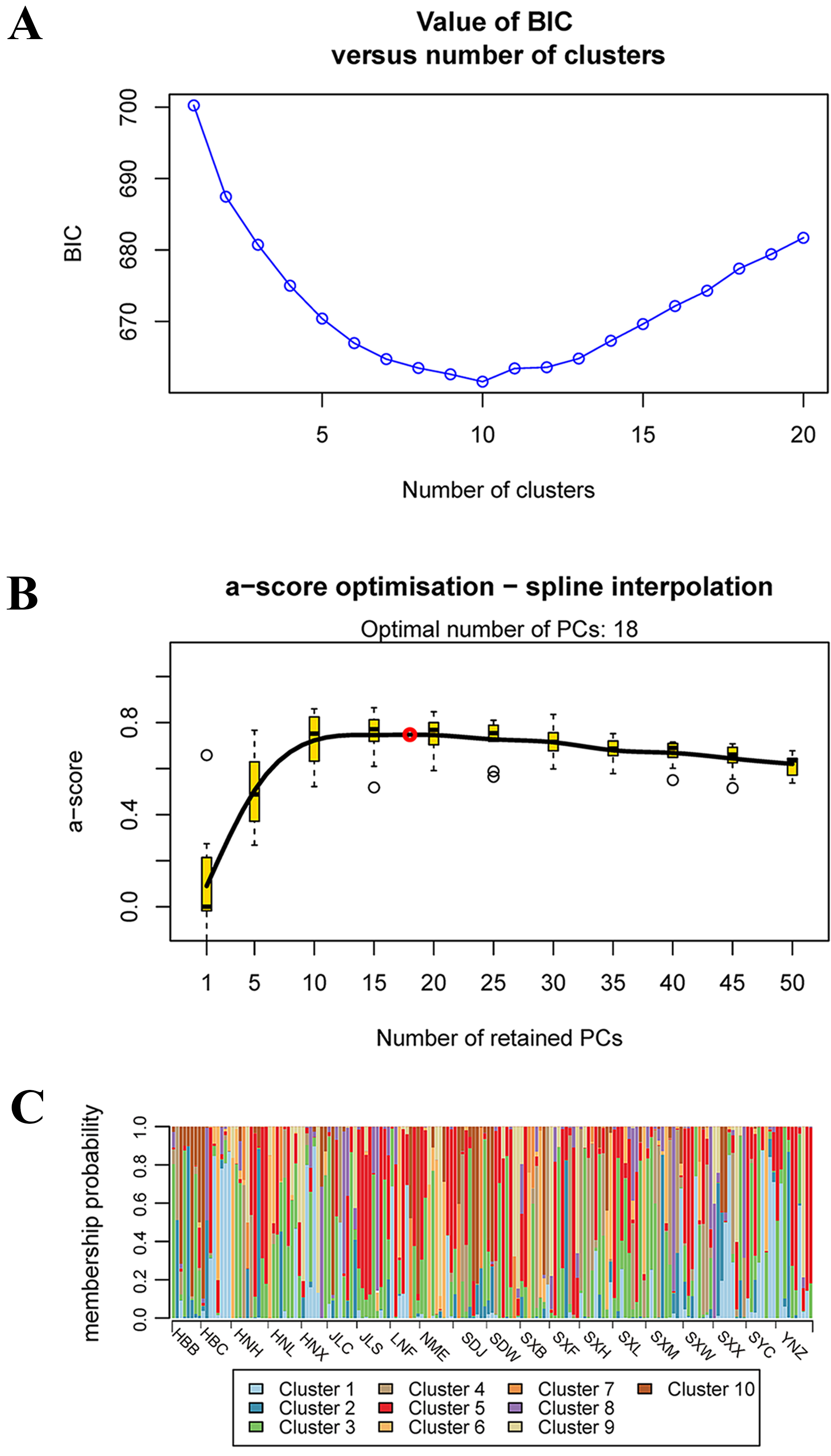

Supplement: Supplementary file 2 [file ECE3-9-13426-s002.tiff]
